# Effects of Bushen-Tiaojing-Fang on the pregnancy outcomes of infertile patients with repeated controlled ovarian stimulation

**DOI:** 10.1038/s41598-021-94366-3

**Published:** 2021-10-11

**Authors:** Yu-Cong Ma, Gui-Min Hao, Zhi-Ming Zhao, Na Cui, Yan-Li Fan, Shuan-Cheng Zhang, Jing-Wei Chen, Yu-Cong Cao, Feng-Li Guan, Jing-Ran Geng, Bu-Lang Gao, Hui-Lan Du

**Affiliations:** 1 Hebei Key Laboratory of Integrative Medicine on Liver-Kidney Patterns, Institute of Integrative Medicine, College of Integrative Medicine, Hebei University of Chinese Medicine, Shijiazhuang, 050091 China; 2 Department of Reproductive Medicine, The Second Hospital of Hebei Medical University, Shijiazhuang, 050000 China

**Keywords:** Biochemistry, Molecular biology, Diseases, Health care, Medical research

## Abstract

Bushen-Tiaojing-Fang (BSTJF) is commonly used to treat infertility. This study investigated the effects of BSTJF on the pregnancy outcomes of patients with repeated controlled ovarian stimulation (COS), on mitochondrial function, and on oxidative stress in ovarian granulosa cells (GCs) and follicular fluid (FF). The samples and clinical data of 97 patients, including 35 in the control group, 29 in the placebo group and 33 in the BSTJF group, were collected for this study. The mitochondrial ultrastructure, ATP content, mitochondrial DNA (mtDNA) number, 8-hydroxy-2-deoxyguanosine (8-OHdG), Mn-superoxide dismutase (Mn-SOD), glutathione peroxidase (GSH-Px) activity levels, and mRNA expression levels of Mn-SOD, GSH-Px, and nuclear factor erythroid-derived factor 2-related factor 2 (Nrf2) were analyzed. The high-grade embryo (*P* < 0.001), implantation (*P* = 0.033), and clinical pregnancy (*P* = 0.031) rates, as well as the ATP content (*P* = 0.014), mtDNA number (*P* = 0.035), GSH-Px activity (*P* = 0.004 in GCs and *P* = 0.008 in FF) and mRNA expression levels (*P* = 0.019), were significantly lower in the placebo group than in the control group, whereas the 8-OHdG content was significantly (*P* = 0.006 in FF) higher in the placebo group than in the control group. Compared with those in the placebo group, the high-grade embryo rate (*P* = 0.007), antioxidant enzyme activity (*P* = 0.037 and 0.036 in Mn-SOD; *P* = 0.047 and 0.030 in GSH-Px) and mRNA level (*P* < 0.001 in Nrf2, *P* = 0.039 in Mn-SOD and *P* = 0.002 in GSH-Px) were significantly higher in the BSTJF group, as were changes in mitochondrial ultrastructure, ATP (*P* = 0.040) and mtDNA number (*P* = 0.013). In conclusion, BSTJF can improve oxidative stress in patients with repeated COS and pregnancy outcomes.

## Introduction

In vitro fertilization and embryo transfer (IVF-ET) is one of the most important methods used to treat infertility. Worldwide, 1.5 million cycles of IVF are carried out every year, resulting in 350,000 IVF babies^1^. As a necessary strategy in IVF-ET, controlled ovarian stimulation (COS) refers to the use of exogenous gonadotropin to promote the development of multiple follicles. However, the success rate of IVF-ET is not as high as expected. The global pregnancy rate per aspiration for fresh nondonor IVF in 2012 was 24.4%, and the pregnancy rates for fresh ICSI and FET were 24.8% and 31.5%, respectively^2^. A study of 451 fertility centers in China showed that the clinical pregnancy rate of each cycle of conventional IVF was 23.2%, that for ICSI cycles was 20.5%, and that for the FET cycle was 48.2%^3^. Thus, to have a successful pregnancy, patients may have to undergo multiple cycles of COS. While this technique continues to evolve, several studies have confirmed some risks associated with this method. Clinical evidence has suggested that fertilization, implantation and pregnancy rates decline with increasing number of COS cycles^4,5^. Experimental studies have shown that repeated COS causes mitochondrial damage in animal reproductive cells, resulting in a decreased mitochondrial DNA copy number, increased DNA fragmentations, an abnormal distribution, and ultrastructural changes^6–9^. Moreover, animal experiments have confirmed that repeated COS increases the reactive oxygen species (ROS) level and 8-hydroxy-2-deoxyguanosine (8-OHdG) and 4-hydroxynonenal (4-HNE) concentrations in the ovaries^10^. Therefore, it is necessary to resolve the issues associated with repeated COS and improve pregnancy outcomes.

During the COS process, the role of ovarian granulosa cells (GCs) and follicular fluid (FF) cannot be ignored. Located in the follicle around oocytes, GCs synthesize and secrete steroid hormones, providing energy support to oocytes. FF is the microenvironment for the growth of GCs and oocytes. Damage to FF can directly affect follicle development and oocyte maturation^11–13^. Oxidative stress is an imbalance between oxidation and antioxidation in the body^14^, and it affects oocyte quality and pregnancy outcomes by damaging ovarian GCs and their mitochondrial functions^15,16^. Although there are some clinical case studies and animal experimental research on repeated COS, the effects on the mitochondrial function and oxidative stress of ovarian GCs in repeated COS patients remain unclear.

At present, Chinese herbal medicine is widely used in IVF-ET in China and has a positive effect on improving the clinical pregnancy rate^17^. Bushen-Tiaojing-Fang (BSTJF) is derived from the ancient famous formula of the Wuzi-Yanzong Pill and Yangjing-Zhongyu Decoction. Clinical research has shown that BSTJF increases the number of oocytes obtained, the number of high-grade embryos, fertilization rates, and clinical pregnancy rates of IVF patients^18–21^. Animal studies confirmed that BSTJF up-regulates the mRNA expression levels of hypoxia-induced factor-1α (HIF-1α) and endothelin 2 (END2), which promotes vasoconstriction and rupture of follicles at the top of follicles, inducing ovulation^22^, up-regulates the protein and mRNA expression levels of vascular endothelial growth factor (VEGF)^23^ and signal transducer and activator of transcription 3 (STAT3) in mouse uteri, improves endometrial receptivity, and promotes embryo implantation^24^. In addition, BSTJF can reduce malondialdehyde (MDA) levels in the ovary tissue of model rats with ovulation disorders and improve total antioxidant capacity (TAC) levels^25,26^. However, its effect on the improvement of ovarian oxidative stress and mitochondrial function in patients with repeated COS is not clear.

In this study, we aimed to explore the effect of repeated COS on clinical pregnancy outcomes, ovarian GCs, FF and mitochondria, to determine whether BSTJF could alleviate the effect of repeated COS and to investigate the molecular mechanism. This study aimed to provide new ideas and experimental bases for the clinical treatment of infertility to improve pregnancy outcomes.

## Results

### Basic characteristics of patients and laboratory indicators

There were no significant differences in age (*P* = 0.158), body mass index (*P* = 0.631), infertility duration (*P* = 0.208), the primary infertility rate (*P* = 0.362), causes of infertility (*P* = 0.984), basic follicle-stimulating hormone (FSH) levels (*P* = 0.445), luteinizing hormone (LH) levels (*P* = 0.235), anti-Mullerian hormone (AMH) levels (*P* = 0.364), or endometrial thickness (*P* = 0.075) on the day of human chorionic gonadotrophin (hCG) injection among the three patient groups. The antral follicular counts (AFCs) were significantly lower in the placebo (11.9 ± 2.7, *P* = 0.037) and BSTJF (11.8 ± 2.4, *P* = 0.015) groups than in the control group (13.3 ± 2.4) (Table 1).

**Table 1:** Clinical characteristics and pregnancy outcome indexes of infertile patients.

Characteristics	Control (n = 35)	Placebo (n = 29)	BSTJF (n = 33)	*P* value
Age (years)	29.7 ± 4.1	30.7 ± 3.2	31.4 ± 3.5	0.158
BMI (kg/m^2^)	23.6 ± 2.8	23.2 ± 2.6	23.9 ± 2.1	0.631
Duration of infertility (years)	3.5 ± 1.2	3.2 ± 1.3	3.8 ± 1.5	0.208
Primary infertility rate (%)	48.6	34.5	51.5	0.362
**Causes of infertility:**				0.984
(a) Tubal factors (%)	57.1	62.1	63.6	–
(b) Male factors (%)	22.9	20.7	18.2	–
(C) Both (%)	20.0	17.2	18.2	–
Basal FSH level (mIU/ml)	6.1 ± 1.1	6.3 ± 1.0	6.4 ± 1.3	0.445
Basal LH level (mIU/ml)	4.3 ± 1.0	4.1 ± 0.8	4.5 ± 1.1	0.235
AMH (ng/ml)	2.8 ± 0.9	2.6 ± 0.9	2.6 ± 0.8	0.364
Antral follicle count	13.3 ± 2.4	11.9 ± 2.7^a^	11.8 ± 2.4^a^	0.033
Endometrial thickness (mm)	11.0 ± 1.7	10.9 ± 1.7	10.8 ± 1.6	0.075
Total Gn dosage (IU)	2435.7 ± 565.3	2663.0 ± 526.7	2462.2 ± 510.4	0.199
No. of retrieved oocytes	13.3 ± 3.8	10.8 ± 2.4^a^	10.1 ± 1.7^a^	0.000
High-grade embryo rate (%)	43.0 (120/279)	25.1 (42/167)^a^	35.7 (76/213)^b^	0.001
Fertilization rate (%)	73.9 (343/464)	70.6 (221/313)	73.6 (245/333)	0.561
Implantation rate (%)	34.3 (23/67)	17.0 (9/53)^a^	29.0 (18/62)	0.101
Clinical pregnancy rate (%)	54.3 (19/35)	27.6 (8/29)^a^	45.5 (15/33)	0.095

One-way analysis of variance, Student’s *t* test and Pearson's chi-square test were used, with the data in mean ± standard deviation or n (%). Control: patients with first-cycle COS; Placebo: repeated COS for 3–5 cycles and treated with placebo; BSTJF: repeated COS for 3–5 cycles and treated with BSTJF.

*n* number of patients, *BMI* body mass index, *AMH* antimullerian hormone, *hCG* human chorionic gonadotrophin, *Gn* gonadotropic hormone, *BSTJF* Bushen-Tiaojing-Fang, *COS* controlled ovarian stimulation.

^a^*P* < 0.05 versus the control group.

^b^*P* < 0.05 versus the placebo group.

There were no significant differences in the total gonadotropin (Gn) dosage (2435.7 ± 565.3 IU, 2663.0 ± 526.7 IU, and 2462.2 ± 510.4 IU, respectively, *P* = 0.199) and fertilization rates (73.9% (343/464), 70.6% (221/313) and 73.6% (245/333), respectively, *P* = 0.561) among the three groups (Table 1). Compared to the control group, the rates of high-grade embryos (43.0% (120/279) vs 25.1% (42/167), *P* < 0.001), implantation (34.3% (23/67) vs 17.0% (9/53), *P* = 0.033), and clinical pregnancy (54.3% (19/35) vs 27.6% (8/29), *P* = 0.031) were all significantly lower in the placebo group. Compared with that in the placebo group, the high-grade embryo rate in the BSTJF group was significantly higher (25.1% (42/167) vs 35.7% (76/213), *P* = 0.007). Although the clinical pregnancy rate of the BSTJF group was higher than that of the placebo group, the difference was not significant (27.6% (8/29) vs 45.5% (15/33), *P* = 0.146).

### Association between predictors and clinical pregnancy outcomes

Each factor was tested in a logistic regression analysis for associations with clinical pregnancy outcomes. The univariate analysis showed that the basal AFC (OR = 1.194, *P* = 0.035), number of retrieved oocytes (OR = 1.197, *P* = 0.012), number of high-quality embryos (OR = 2.180, *P* = 0.000), and fertilization rate (OR = 1.422, *P* < 0.001) were significantly associated with clinical pregnancy outcomes. However, multivariate regression analysis of these predictors revealed that only the number of high-quality embryos was significantly (OR = 1.977, *P* = 0.001) associated with clinical pregnancy outcomes (Table 2).

**Table 2:** Univariate and multivariate regression analysis.

Variables	Univariate			Multivariate		
	OR	95% CI	*P* value	OR	95% CI	*P* value
Antral follicle count	1.194	1.012–1.408	0.035	0.940	0.739–1.195	0.614
No. of retrieved oocytes	1.197	1.040–1.377	0.012	0.910	0.711–1.164	0.451
No. high-grade embryo	2.180	1.540–3.086	0.000	1.977	1.308–2.986	0.001
No. of fertilization	1.422	1.183–1.709	0.000	1.299	0.984–1.715	0.065

*OR* odds ratio, *AFC* antral follicle count in both ovaries, *CI* confidence interval.

### Observation of mitochondrial ultrastructure

**Figure 1:**
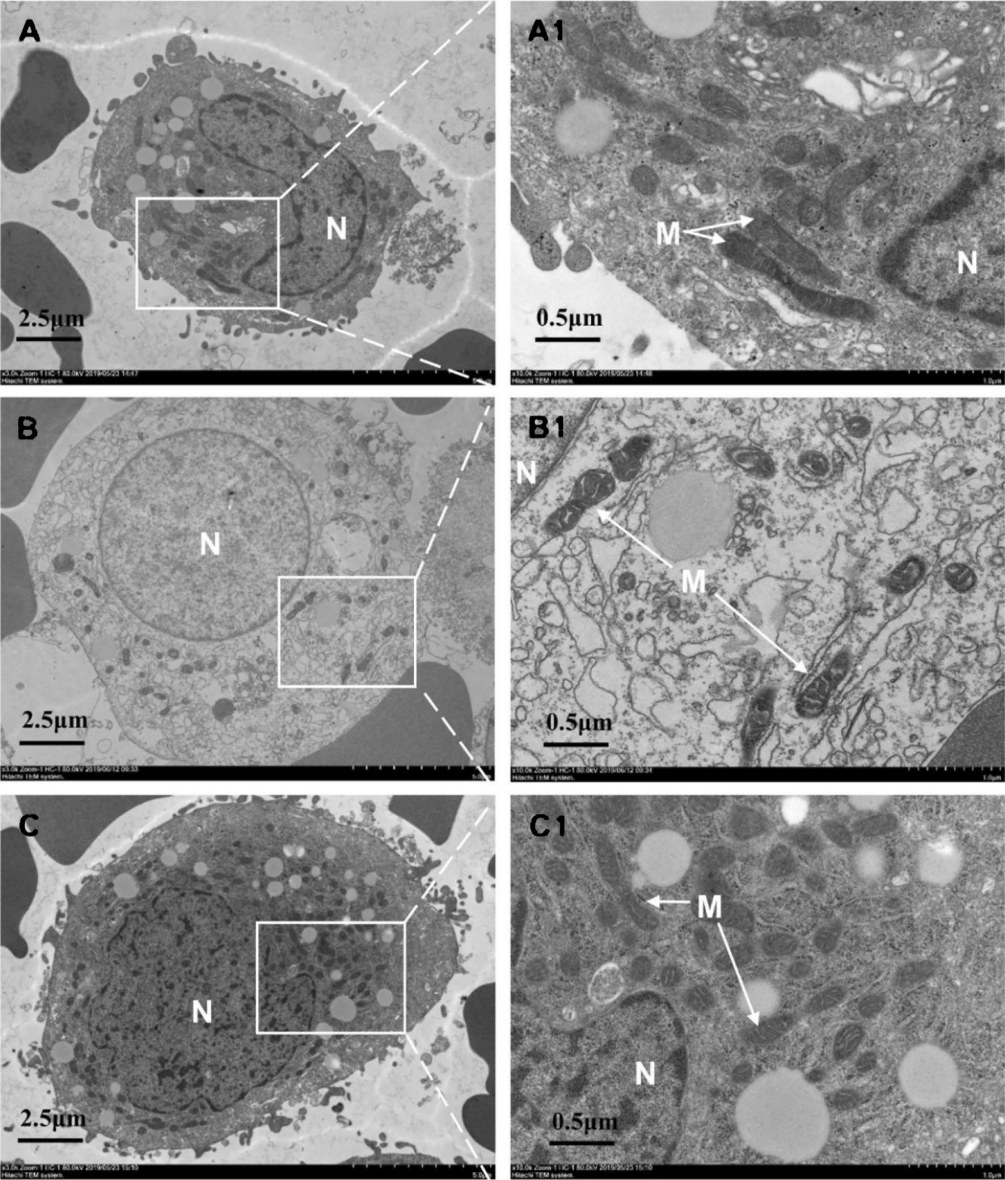
Comparison of mitochondrial ultrastructure in ovarian GCs. (**A**–**C**) The control, placebo and BSTJF group, respectively, whereas (**A1**–**C1**) the local magnification in (**A**–**C**), respectively. Scale bars are 2.5 μm in (**A**–**C**) but 0.5 μm in (**A1**–**C1**); *N* nucleus, *M* mitochondria, *GCs* granulosa cells, *BSTJF* Bushen-Tiaojing-Fang.

The effects of repeated COS and BSTJF on the ultrastructure of GC mitochondria were investigated. Significantly different mitochondrial structures were found between the three groups. In the control group, the mitochondria were round or rod-shaped with normal sizes, a dense distribution, and clear structures (Fig. 1A, A1). In the placebo group, the mitochondria were atrophied, with a dense matrix, an expanded cristae cavity and vacuoles, and a decreased number of cristae (Fig. 1B, B1). In the BSTJF group, the mitochondria were abundant, with only a slightly expanded inner ridge (Fig. 1C, C1).

### Mitochondrial ATP content and mitochondrial DNA (mtDNA) copy number in ovarian GCs

**Figure 2:**
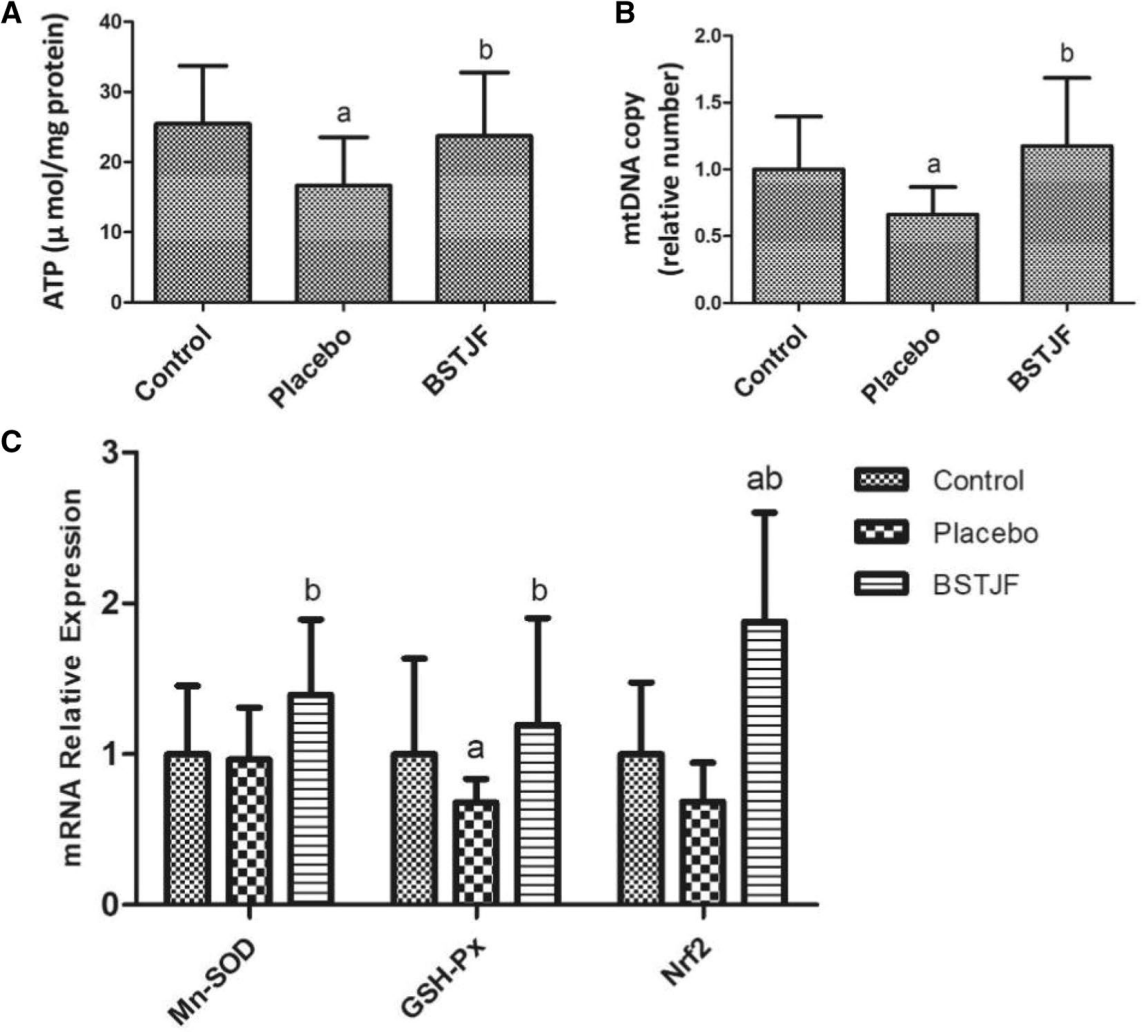
Comparison of mitochondrial ATP content, mitochondrial DNA (mtDNA) copy number, and Nrf2 mRNA expression levels in ovarian GCs. (**A**) Comparison of the mitochondrial ATP content. (**B**) Comparison of mitochondrial DNA copy number. (**C**) Nrf2 mRNA expression levels in ovarian GCs. ^a^*P* < 0.05 versus the control group; ^b^*P* < 0.05 versus the placebo group; Student’s *t* test was used to compare the two groups. *GCs* granulosa cells, *BSTJF* Bushen-Tiaojing-Fang.

Compared to that in the control group, the mitochondrial ATP content in the placebo group was significantly lower (*P* = 0.014), but that in the BSTJF group was not significantly lower (*P* = 0.615). The ATP content was significantly higher in the BSTJF group than in the placebo group (*P* = 0.040) (Fig. 2A). Similarly, the concentration of mtDNA in the placebo group was significantly lower than that in the control group (*P* = 0.035), whereas the number of mtDNA in the BSTJF group was significantly higher than that in the placebo group (*P* = 0.013) (Fig. 2B).

### 8-OHdG content in ovarian GCs and FF

**Figure 3:**
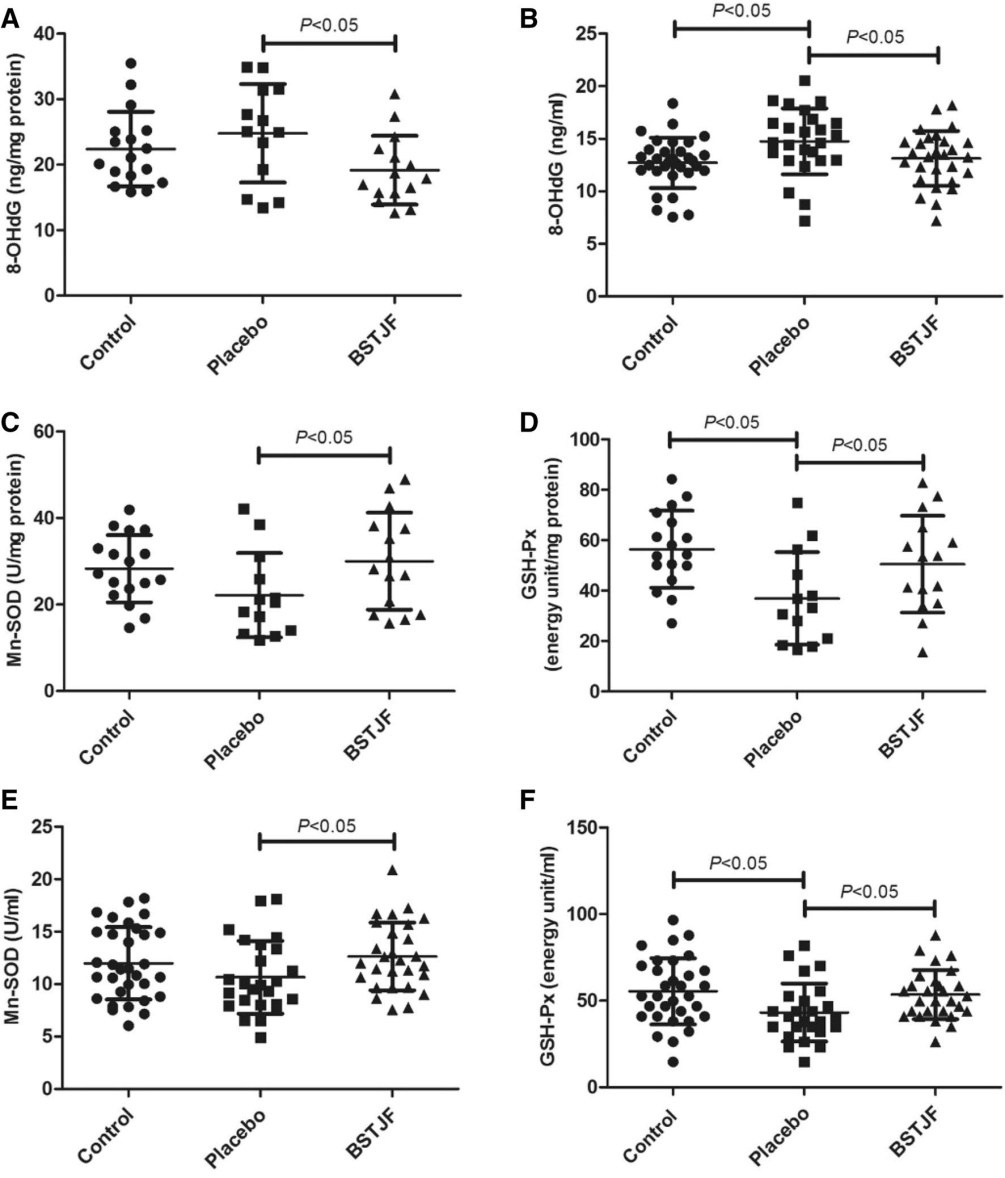
Comparison of oxidative stress markers in the ovarian GCs and FF. **A**, B 8-OHdG levels in GCs and FF. Antioxidant enzymes Mn-SOD and GSH-Px activity levels in the ovarian GCs (**C**,**D**) and FF (**E**,**F**). ^a^*P* < 0.05 versus the control group; ^b^*P* < 0.05 versus the placebo group; Student’s *t* test was used to compare the two groups. *GCs* granulosa cells, *FF* follicular fluid.

The 8-OHdG levels in ovarian GCs were not significantly (*P* = 0.290) different between the placebo group and the control group (Fig. 3A), but 8-OHdG levels in FF were significantly higher in the placebo group than in the control group (*P* = 0.006) (Fig. 3B). The levels of 8-OHdG in both GCs (*P* = 0.020) (Fig. 3A) and FF (*P* = 0.032) (Fig. 3B) in the BSTJF group were significantly lower than those in the placebo group but were not significantly different from those in the control group (*P* = 0.147 and *P* = 0.544).

### Mn-superoxide dismutase (Mn-SOD) and glutathione peroxidase (GSH-Px) activity in ovarian GCs and FF

Mn-SOD and GSH-Px activity in ovarian GCs (Fig. 3C,D) and FF (Fig. 3E,F) was tested by ELISA. A significant difference was found in the GSH-Px level (*P* = 0.004 and *P* = 0.008, respectively) (Fig. 3D,F) but not in the Mn-SOD level (*P* = 0.092 and *P* = 0.145, respectively) (Fig. 3C,E) between the placebo and control groups, indicating that the antioxidant enzyme activity was lower with repeated COS. However, in the ovarian GCs (Fig. 3C,D) and FF (Fig. 3E,F), the BSTJF group had higher levels of both Mn-SOD (*P* = 0.037 and *P* = 0.036, respectively) and GSH-Px (*P* = 0.047 and *P* = 0.030, respectively) than the placebo group, suggesting that BSTJF could improve antioxidant capacity in patients with repeated COS.

### Mn-SOD, GSH-Px, and nuclear factor erythroid-derived factor 2-related factor 2 (Nrf2) mRNA in ovarian GCs

The mRNA expression levels of Mn-SOD, GSH-Px, and the upstream regulator Nrf2 were tested. The mRNA level of GSH-Px was significantly lower (*P* = 0.019) in the placebo group than in the control group, and Mn-SOD and Nrf2 showed a higher trend in the placebo group than in the control group, but the difference was not significant (*P* = 0.856 and *P* = 0.265, respectively). However, administration of BSTJF significantly increased not only the Nrf2 mRNA expression level (*P* < 0.001) compared with the control group but also the Mn-SOD (*P* = 0.039) and GSH-Px levels (*P* = 0.002) compared with the placebo group (Fig. 2C).

## Discussion

Our study demonstrated that, compared to the first COS in the control patients, patients with repeated COS had a significantly lower number of retrieved oocytes and significantly lower rates of high-grade embryos, implantation, and clinical pregnancy, in addition to increased damage of the mitochondrial ultrastructure, a lower ATP content and a lower mtDNA number in ovarian GCs. Moreover, patients with repeated COS had a higher 8-OHdG content, lower Mn-SOD and GSH-Px activity and lower Mn-SOD, GSH-Px, and Nrf2 mRNA expression. Compared to the placebo group, the BSTJF group had a significantly higher high-grade embryo rate among patients with repeated COS and an improved abnormal mitochondrial ultrastructure, ATP content, mtDNA number, antioxidant enzyme activity and mRNA expression levels.

Compared with the first COS in the control group, repeated COS did not significantly affect basic FSH and AMH levels, but the AFC, another indicator of ovarian reserve^27^, was significantly lower. In addition, the number of retrieved oocytes, high-grade embryo rate, implantation rate and clinical pregnancy rate were significantly lower among patients with repeated COS. Clinical studies have shown that the success rate of IVF-ET decreased with increased numbers of COS cycles^28,29^. Most studies have demonstrated that COS with 1–2 cycles has no significant effect on ovarian reserve, oocyte number, or the clinical pregnancy rate, whereas COS with 3 or more cycles can significantly reduce the clinical pregnancy rate^5,30,31^, even though repeated COS has been reported to not affect the number of oocytes, embryo quality, or pregnancy outcome^32^. Our studies of patients with first COS and repeated COS (3–5 cycles) confirmed that repeated COS had adverse effects on the patient pregnancy outcomes, consistent with the findings of most studies.

Administration of BSTJF significantly (*P* = 0.007) increased the high-grade embryo rate compared with the placebo. The clinical pregnancy rate was also higher after administration of BSTJF, even though it was not significantly different from that in the placebo group (this lack of significance may be due to the small number of patients). Logistic regression analyses revealed that the number of high-quality embryos was significantly related to the clinical pregnancy outcome and could be used as an important indicator to predict pregnancy outcomes. This suggests that BSTJF can improve the pregnancy outcome by improving embryo quality in patients with repeated COS. In normal healthy women, only one egg is typically discharged every month; however, in repeated COS, drugs are used to promote the development of multiple follicles at the same time. This may lead to poor follicle development and a low pregnancy rate. Herbs such as *Radix Rehmanniae Preparata*, *Cornus officinalis*, *Fructus Lycii*, *Semen Cuscutae*, and *Raspberry* in BSTJF are commonly used in traditional Chinese Medicine and can invigorate health and restore reproductive function^33–35^, and the effective components of these drugs, such as catalpa, morroniside, and oleanolic acid, can increase the activity of Mn-SOD and GSH-Px but reduce the level of ROS^33–35^. This may be the reason why these herbs and their active ingredients in BSTJF can improve the high-quality embryo rate and pregnancy outcomes.

In our study, repeated COS (in the placebo group) was shown to cause abnormal changes in the ultrastructure of mitochondria in ovarian GCs, and ATP content and mtDNA number were significantly lower than those in the first-cycle COS (control) group. The mitochondria in ovarian GCs are very important for the development and maturation of oocytes, as well as for oocyte quality and embryonic development^12,36^. Most of the energy that oocytes require is provided by GCs, and the ATP of GCs can be directly transmitted to oocytes through the gap of cumulus GCs^37,38^. Repeated COS can result in a reduction in the mitochondrial DNA copy number in mouse GCs^8^ and degradation of mitochondrial cristae in rhesus monkeys^9^, which directly affects the production of ATP. Our results agreed with the above studies. In addition, mitochondrial dysfunction of GCs leads to poor oocyte quality and lower female fertility^39,40^, which also explains our finding that patients with repeated COS had poor pregnancy outcomes. It is worth noting that the mitochondrial ultrastructure, ATP content, and mtDNA number in the BSTJF group were significantly higher than those in the placebo group, suggesting that BSTJF can improve mitochondrial function. This may be one reason why the rate of high-quality embryos in the BSTJF group was higher than that in the placebo group.

Under physiological conditions, mitochondria have their own ROS clearance systems. However, mitochondria, as the site of oxidative phosphorylation and ATP synthesis, are also most vulnerable to ROS attacks. Excessive production of ROS is associated with changes in mitochondrial membrane potential and DNA damage in ovarian GCs^15^. 8-OHdG is a hallmark product of DNA damage caused by oxidative stress. Repeated COS can increase the 8-OHdG level in mice^10^, and a high level of 8-OHdG in ovarian GCs is associated with a low fertilization rate of oocytes and poor embryo quality during IVF^41^. Our study confirmed that the ovarian GCs and FF of patients with repeated COS (placebo group) did show higher levels of 8-OHdG. Moreover, high levels of SOD and GSH-Px activity were related to the success rate of IVF^42,43^, which suggested that poor clinical pregnancy outcomes and mitochondrial function damage were associated with the imbalance of oxidation and antioxidation caused by repeated COS. However, administration of BSTJF in our study significantly decreased the levels of 8-OHdG but increased the Mn-SOD and GSH-Px levels compared to the placebo group, indicating that BSTJF can improve mitochondrial function and clinical pregnancy outcomes by adjusting the oxidation-antioxidation balance.

Our study also showed that in patients with repeated COS, the mRNA expression levels of Mn-SOD, GSH-Px, and Nrf2 in ovarian GCs were significantly lower than those in patients with first COS; however, administration of BSTJF could significantly upregulate the Nrf2 mRNA expression level as well as the expression of Nrf2 target genes including Mn-SOD and GSH-Px. The Nrf2 system is considered to be an important antioxidant stress mechanism^44^. When confronted with various stimuli, Nrf2 enters the nucleus and forms a complex with the maf protein and antioxidant response elements (AREs) to initiate the transcription of antioxidant enzymes such as SOD and GSH-Px^45^. Many studies have confirmed that the active monomer components, including catalpol, oleanolic acid, and quercetin, in BSTJF can upregulate Nrf2 expression^46–48^, and our study confirmed the significant upregulation of the mRNA levels of Nrf2 by BSTJF. Thus, BSTJF plays an antioxidant and protective role by reducing the attack of ROS on mitochondria to protect the normal function of mitochondria. This may be one of the mechanisms by which BSTJF improves the clinical pregnancy outcome.

Our study had some limitations, including a small cohort of patients. Moreover, the mechanism by which BSTJF upregulates Nrf2 should be investigated to better understand the clinical role of BSTJF. The single-center nature of this study and its enrollment of only Chinese patients may have led to the publication bias. These limitations will have to be addressed in future studies for better outcomes.

In summary, BSTJF can increase the mRNA expression of Mn-SOD and GSH-Px by upregulating the Nrf2 signaling pathway, and then improve oxidative stress and mitochondrial function, thus increasing the high-grade embryo rate of repeated COS patients. This study may provide new evidence for the use of BSTJF in the treatment of infertility and improvement of IVF-ET pregnancy outcomes.

## Methods

### Preparation and composition analysis of BSTJF

BSTJF consists of *Radix Rehmanniae Preparata (Shu-di)* 20 g, *Cornus officinalis (Shan-Zhu-yu)* 15 g, *Rhizoma Dioscoreae (Shan-yao)* 12 g, *Fructus Lycii (Gou-Qi-zi)* 12 g, *Semen Cuscutae (Tu-Si-zi)* 12 g, *Raspberry (Fu-Pen-zi)* 10 g, *Cistanche deserticola (Rou-Cong-rong)* 10 g, *Placenta Homini (Zi-He-che)* 10 g, *Fructus Ligustri Lucidi (Nv-Zheng-zi)* 9 g, *Radix Angelicae Sinensis (Dang-gui)* 9 g, and *Radix Paeoniae Alba (Bai-shao)* 9 g. The BSTJF decoction granules were purchased from Shineway Pharmaceutical Group Co., Ltd. (Shijiazhuang, China). The granules were dissolved in 200 ml of water, and 200 μl sample was added and mixed with 1 ml of 80% methanol. The mixture was centrifuged at 12,000*g* for 10 min at 4 °C. The supernatant was filtered with a 0.22 μm filter and analyzed by chromatography (UltiMate 3000 RS, Thermo Fisher Scientific, USA) and Q Exactive high-resolution mass spectrometry (Thermo Fisher Scientific, USA).

**Figure 4:**
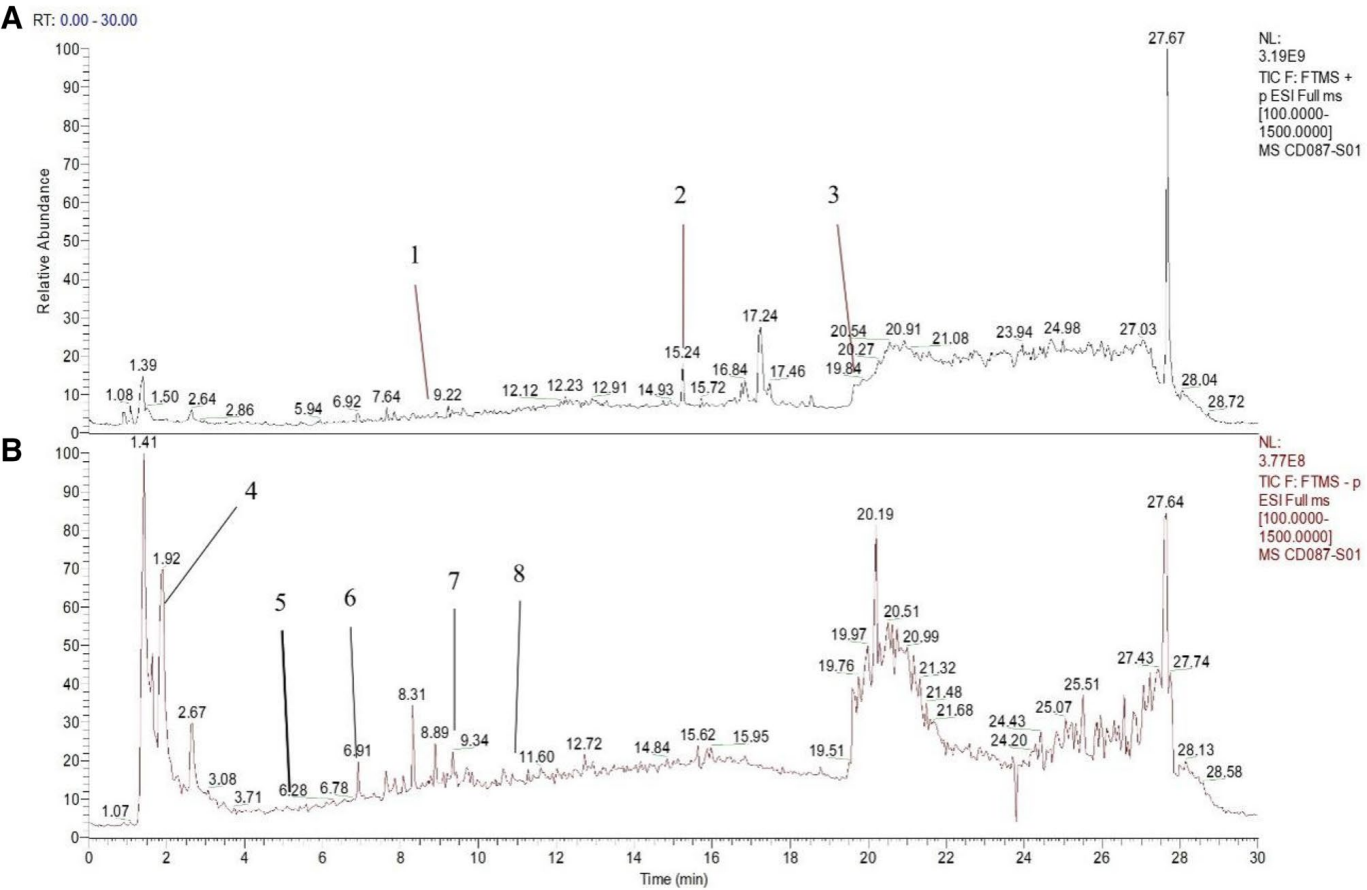
Total ion flow pattern of the antioxidants in Bushen-Tiaojing-Fang. (**A**) Positive ion mode; (**B**) negative ion mode.

Mass spectrometry was performed using the following conditions: electrospray ionization source, positive and negative ion switching scanning, detection method of Full mass/dd-MS_2_, resolution 70,000/17,500, scanning range 100–1500 *m*/*z*, electrospray voltage 3.8 kv (positive), capillary temperature 300 °C, high purity argon as the collision gas, nitrogen (40Arb) as the sheath gas, nitrogen (350 °C) as the auxiliary gas, and a data collection time of 30 min. Chromatographic conditions were as follows: chromatographic column (PR-C18, 150 × 2.1 mm, 1.8 μm, Welch), flow rate 0.3 ml/min, aqueous 0.1% formic acid solution, organic 0.1% formic acetonitrile, methanol as the washing solution, column temperature 35 °C, automatic sample injector 10 °C, injection needle height of 2 mm, volume of washing needle of automatic sample injector of 200 μl, soaking time of 3 ms during cleaning, and sample volume of 10 μl. Collected data were preliminarily processed using CD2.1 (Thermo Fisher Scientific, USA) and then compared with Database Retrieval (mzCloud, mzVault, ChemSpider). We detected eight compounds (including rutin, ligustilide, oleanolic acid, verbascoside, catalpol, morroniside, astragalin and quercetin) from BSTJF that have been confirmed to have antioxidant effects^34,35,49–54^. Details of these components are shown in Table 3 and Fig. 4.

**Table 3:** Eight antioxidant components of ions identified in Bushen-Tiaojing-Fang.

No	Retention time (min)	Compound	Molecular formula	Molecular weight	Charged property	MS1	MS2
1	8.69	Rutin	C27H30O16	610.15	+	611.16	303.05
2	15.20	Ligustilide	C12H14O2	190.24	+	191.11	173.10
3	19.52	Oleanolic acid	C30H48O3	456.36	+	439.36	203.18
4	2.07	Verbascoside	C29H36O15	624.59	−	624.24	163.04
5	5.05	Catalpol	C15H22O10	362.33	−	361.08	169.01
6	6.85	Morroniside	C17H26O11	452.00	−	451.15	243.09
7	9.34	Astragalin	C20H20O11	448.10	−	447.09	284.03
8	10.99	Quercetin	C15H10O7	302.00	−	301.04	151.00

*MS1* primary parent ion, *MS2* second ions which are the characteristic fragments of MS1 after fragmentation by mass spectrometry.

### Subjects

This study was approved by the Ethics Committee of Hebei University of Chinese Medicine (Shijiazhuang, China), and all patients signed informed consent to participate. All methods were performed in accordance with the relevant guidelines and regulations. The inclusion criteria were patients who experienced IVF-ET due to infertility caused by tubal factors or male factors (varying degrees of oligospermia, asthenia, and teratozoospermia, but not azoospermia) or both, with an age ≤ 38 years, regular menstruation (cycle 21–35 days, period 2–8 days, menstrual volume 20–60 ml)^55^, basic FSH < 10 IU/L, first-cycle COS or repeated COS for 3–5 cycles. The exclusion criteria were the same as those in the previous study^56^: patients who were unsuitable for pregnancy due to conditions such as uterine abnormalities or serious heart diseases and who suffered from serious mental disorders, urinary and reproductive system infection, sexually transmitted diseases, endometriosis, uterine fibroids, tuberculosis, uterine adhesion, endometrial lesions, polycystic ovary syndrome, ovarian cyst, and hydrosalpinx. Patients who had been exposed to teratogenic radiation or poison and who were within the action period of the drugs were also excluded.

**Figure 5:**
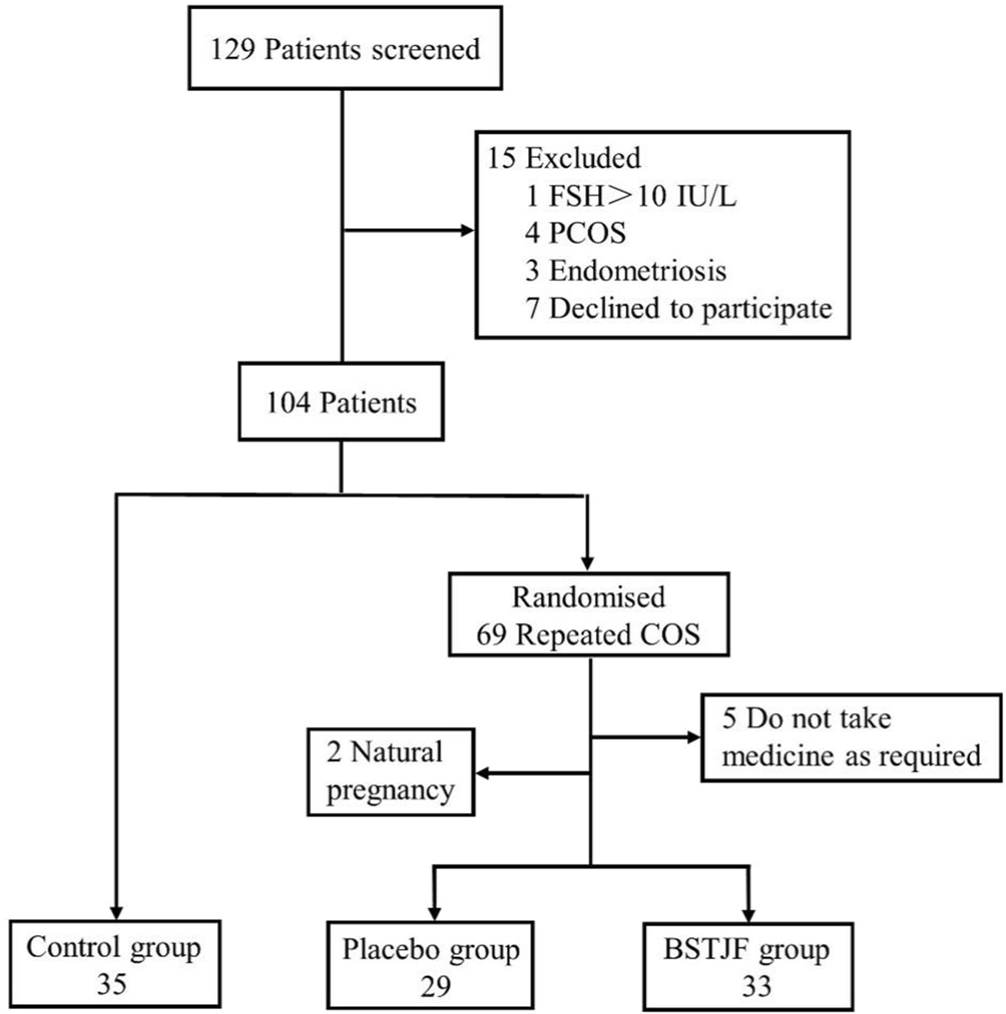
Flow diagram of inclusion of patients in the groups. *COS* controlled ovarian stimulation, *BSTJF* Bushen-Tiaojing-Fang.

In total, 129 patients were recruited from January 2018 to April 2019, among which 15 patients were excluded (including patients who did not meet the inclusion criteria, met the exclusion criteria, and refused to participate). In total, 104 eligible patients were included, of whom 35 with their first COS cycle were included the control group, and the remaining 69 patients with repeated COS were randomly divided into the placebo group and the BSTJF group by SPSS 21.0 software (IBM, Chicago, IL, USA). Specific steps are as follows: According to the number of subjects enrolled in the plan (78 cases were included in the plan, but 69 cases were actually included), a series of serial numbers arranged from 1 to 78 were created in SPSS 21.0 software as the serial number of subjects enrolled. The random seed was set as 20,170,912. Uniform random number generation function Rv. Uniform was used to generate a random number for each serial number. The first 38 random numbers are divided into group A and the last 38 into group B according to the size of random numbers. When using, the grouping results are determined according to the order of subjects' inclusion, so as to realize the randomization of subjects. The drug number is the random number, which is the above-mentioned sequence number. The corresponding drugs are packaged according to the random results, and the study drugs are distributed according to the order of patient enrollment. The patients with repeated COS took the drug for 3 months before the next COS cycle. Seven patients dropped out during this process (5 were unable to maintain their medication, and 2 became pregnant naturally). Finally, GC, FF, and clinical data were collected from 29 patients in the placebo group and 33 patients in the BSTJF group (Fig. 5).

### IVF process

All patients were treated with the GnRH antagonist protocol. Recombinant FSH (Gn, Gonal-F; Merck Serono; Merck KGaA, Darmstadt, Germany) was administered on the third day of menstruation to promote follicular growth. The Gn dosage was adjusted according to the follicle conditions under B-ultrasonography monitoring and the serum estrogen levels. Antagonists (Cetrorelix; Merck Serono; Merck KGaA, Darmstadt, Germany) were added when 5–6 follicles were 10–13 mm in diameter or when the LH level was over 10 mIU/ml. After 8–14 days, 250 μg of hCG (hCG; Merck Serono; Merck KGaA) was injected at night when the diameters of at least 2–3 follicles reached 18 mm in both ovaries and the number of follicles above 14 mm matched the serum estrogen level. The oocytes were harvested via ultrasound-guided transvaginal puncture oocyte retrieval surgery 36–38 h later. Oocytes were picked up and placed in incubators for fertilization. FF was placed in sterile enzyme-free centrifuge tubes for further separation and detection.

On the third day after fertilization, embryo morphology and cell number were observed to determine the embryo quality. According to the standards of the Istanbul conference^57^, a grade I embryo indicates a day-3 two pronuclear (2PN) embryo with 7–9 cells, a uniform cell size, and a fragmentation rate of 10% or less, and a grade II embryo has a number of blastomeres that is consistent with the developmental stage of the embryo and has a fragmentation rate between 11 and 25%. In grade III-IV embryos, the number of blastomeres is not consistent with the developmental stage, and the fragmentation rate is > 25%, with vacuoles and multinucleation. Grade I–II embryos were considered of high quality. One or two embryos were transferred to each patient. Four weeks after transplantation, a pregnancy sac confirmed by ultrasound was considered a clinical pregnancy. The implantation rate was defined as the ratio of the gestational sac number to the number of embryos transferred.

### Extraction of ovarian granulosa cells

The FF and GC suspensions were collected and centrifuged at 4 °C (433×*g*, 10 min). The FF supernatant was absorbed and stored in a − 80 °C freezer until further use. PBS was added into the sediment to 5 ml and mixed. Five milliliters of human lymphocyte separation fluid (Lympholyte-H, Cedarlane Laboratories, Canada) was added to another centrifuge tube, and PBS suspension was slowly added to the surface of the human lymphocyte separation solution for centrifugation at 4 °C (680×*g*, 10 min). GCs were extracted, and 200–300 μl of 0.2% hyaluronidase (H8030, Beijing Solarbio Science and Technology Co., Ltd., Beijing, China) was added. After blowing and digestion for 3 min, 1 ml of PBS was added to terminate the digestion, and centrifugation was performed at 4 °C (982×*g*, 5 min). GCs were divided into several parts and stored in a—80 °C freezer until use.

### Mitochondria ultrastructure

Electron microscopy stationary liquid (G1102, Servicebio, China) was rapidly added to the extracted ovarian GC sample. After 4 h of fixation at 4 °C, the cells were rinsed 3 times with phosphate buffer (pH7.4). Then, the GC sample was placed in 1% osmic acid, fixed at room temperature for 2 h, and rinsed with phosphate buffer (pH 7.4) 3 times. Next, the GC sample was dehydrated and embedded in ethyl alcohol and acetone at different concentrations. Afterwards, the GC sample was sliced into 50 nm ultrathin sections using an ultrathin slicer (EM UC7, Leica) and stained with uranyl acetate and lead citrate. Finally, the GC mitochondria were observed using a transmission electron microscope (HT7700, Hitachi, Japan).

### Mitochondrial adenosine triphosphate (ATP)

Mitochondrial ATP content was detected using an ATP detection kit (S0027, Beyotime Institute of Biotechnology, China). A total of 200 μl of lysate was added to each tube of ovarian GCs sample for centrifugation at 12,000*g* for 5 min. The supernatant was removed for future use. The standard solution and test working solution were prepared according to the instructions. Then, the sample supernatant, standard solution, and test working solution were added to a 96-well plate according to the instructions. The readings were made using Fusion FX5 Spectra (Vilber Lourmat, Marne-la-Vallée, France), and the concentrations were calculated according to the standard curve concentration. To boost accuracy, the protein concentrations of each group of samples were determined by a BCA protein concentration assay kit (PC0020, Solarbio, China), and the ATP content was converted to μmol/mg protein.

### ELISA of 8-OHdG, Mn-SOD, and GSH-Px

The kits used for the detection of Mn-SOD and GSH-Px were purchased from Jiancheng Bioengineering Institute (Nanjing, China), and 8-OHdG was detected using 8-hydroxy-2 deoxyguanosine ELISA kits (ab201734, Abcam, USA). The ovarian GC sample lysis fluid was diluted to the optimal concentration to meet the standard curve for detection. The absorbance values were measured by enzyme-linked immunoassay (VersaMax, Molecular Devices, USA). A BCA protein concentration assay kit (PC0020, Solarbio, China) was used to determine the protein concentration.

### Real-time fluorescence quantitative PCR

The mRNA expression levels of Mn-SOD, GSH-Px, and nuclear factor erythroid-derived factor 2-related factor 2 (Nrf2) in GCs were determined using qRT-PCR. Briefly, RNA was extracted using TRIzol (Cat No. 15596026, Invitrogen, USA) and reverse transcribed into cDNA using a reverse transcription system (R101-01/02, Vazyme BioTech, Nanjing, China). Target RNA was amplified using a real-time fluorescence quantitative PCR system (Q111-02, Vazyme BioTech, Nanjing, China). Mitochondrial DNA (mtDNA) was amplified using a FastStart Essential DNA Green Master kit (06,402,712,001, Roche, Switzerland). Specific primer sequences (designed and synthesized by Wuhan Qingke company and Suzhou Genewiz Biotechnology Co., Ltd., China) are shown in Table 4. The final data were analyzed using 2^−ΔΔCt^.

**Table 4:** List of the primers used in the qRT-PCR analysis.

Genes	Primers	Amplified size (bp)
Mn-SOD	CCCGACCTGCCCTACGACTA CTCCCCTTTGGGTTCTCCAC	264
GSH-Px	CCAGTCGGTGTATGCCTTCT CGTTCTCCTGATGCCCAAAC	221
Nrf2	GTCAGCGACGGAAAGAGTA GTGGGCAACCTGGGAGTAG	203
GAPDH	TCAAGAAGGTGGTGAAGCAGG TCAAAGGTGGAGGAGTGGGT	115
Mitochondria COII	CCCCACATTAGGCTTAAAAACAGAT TATACCCCCGGTCGTGTAGCGGT	100
Nucleus GAPDH	GTCAGCCGCATCTTCTTTTG GCGCCCAATACGACCAAATC	80

### Statistical analysis

SPSS 21.0 software (IBM, Chicago, IL, USA) was used for data analysis. Continuous variables were tested for normality using the Shapiro–Wilk test. One-way analysis of variance and Student’s t-test were used for data conforming to a normal distribution. The Pearson chi-square test was used to compare the rates between samples. The data are presented as the mean ± standard deviation (SD) or n (%). Logistic regression analyses were performed to determine the association between various factors (including AFC, number of retrieved oocytes, high-grade embryo number and fertilization number) and the clinical pregnancy outcome, with the odds ratio (OR) value (95% confidence interval (CI)) representing the degree of correlation*. P* < 0.05 was considered statistically significant.

## Data Availability

The data sets used and/or analyzed during the current study are available from the corresponding author on reasonable request.
